# Sexual and reproductive health rights knowledge and reproductive health services utilization among rural reproductive age women in Aleta Wondo District, Sidama zone, Ethiopia: community based cross-sectional study

**DOI:** 10.1186/s12914-020-00223-1

**Published:** 2020-03-11

**Authors:** Tariku Tadesse, Mesay Hailu Dangisso, Teshome Abuka Abebo

**Affiliations:** 1 Aleta Wondo District Health Office, Aleta Wondo, Sidama, Ethiopia; 2 School of Public Health, College of Medicine and Health Sciences, Hawassa University, Hawassa, Ethiopia

**Keywords:** Sexual, Reproductive health, Rights

## Abstract

**Background:**

Various countries in the world have achieved promising progress in promoting, protecting and guaranteeing sexual and reproductive health rights (SRHRs) since the 1994 International Conference on Population and Development (ICPD) in Cairo. However, SRHRs have not been recognized to their maximum potential in Ethiopia, despite the domestication of the international instruments related to their successful implementation. This study was intended to determine the magnitude of SRHRs knowledge, reproductive health services utilization and their independent predictors among rural reproductive-age women in the Aleta Wondo District, Ethiopia.

**Methods:**

A community-based cross-sectional study was conducted among 833 rural reproductive-age women from April to May 2019. A systematic random sampling technique was employed to select households, and a structured questionnaire was used to gather the data. EPI INFO version 7 was used to enter the data, and SPSS version 23 was used for data analysis. Logistic regression analysis was employed to assess the association between outcomes and explanatory variables. Odds ratios at 95% CI were also computed and reported.

**Results:**

Of 833 respondents, 43.9% had good knowledge of SRHR, and 37% had used at least one sexual and reproductive health (SRH) service. Variables that had a statistically significant association with SRHR knowledge in multivariable analysis were: had formal education, household with the highest income, having information sources for SRH services, and knowing about SRH services and providing institutions. SRH services utilization was associated with: having information sources for SRH services, had formal education, household with the highest income, and knowing about SRH services and providing institutions.

**Conclusion:**

In this study demographic and economic factors, such as education and household monthly income were positively identified as independent predictors for knowledge of SRHR and SRH services utilization. Therefore, responsible government sectors and NGOs should design and implement programs to promote women’s educational status and household economic status to enhance women’s SRHR knowledge and SRH services utilization.

## Background

Sexual and reproductive health rights (SRHR) are fundamental to people’s health and survival, to economic development, and the wellbeing of humanity. The global health and human rights communities have proactively worked for decades to define and advance SRHR, encountering both advances and considerable setbacks [1, 2]. Reproductive rights were succinctly described at ICPD as resting on, “the basic right of all couples and individuals to decide freely and responsibly the number, spacing, and timing of their children and to have the information and means to do so.” Reproductive health rights and sexual rights are invariably human rights recognized by various human rights instruments. Sexual rights offer individuals the opportunity to freely choose partners without any form of discrimination and freedom to assert on and exercise safer sex [2, 3].

The Millennium Development Goals (MDGs) in 2000, health and development initiatives including the 2030 Agenda for Sustainable Development, and the movement toward universal health coverage further support the realization of the reproductive health rights that were already recognized in the ICPD in 1994 [4]. The Ethiopian government has responded to international conventions and human rights treaties like ICPD, MDGs, and SDGs by reviewing its laws and policies. One of the responses was the development of the National Reproductive Health Strategy to promote utilization of SRH services and information, reduce gender-based violence and harmful traditional practices [5]. Article 35 of the country’s constitution refers to women’s equality with men and their rights to information and the right to be protected from the dangers of pregnancy and childbirth [6]. The Ministry of Health designed a five-year Health Sector Transformation Plan (HSTP) that devotes special attention to maternal health service utilization to reduce maternal mortality through implementation of high impact interventions like antenatal care (ANC), skilled birth services and postnatal care (PNC), women’s empowerment, gender mainstreaming, abortion care, fistula care, adolescent and reproductive health care [5, 7].

In Ethiopia, reproductive health service quality and access are among the major public health challenges. A recent nationwide survey revealed that 41% of currently married women are utilizing modern contraceptive methods. Also the survey results show that 74% of women who gave birth in the 5 years preceding the survey received antenatal care from a skilled provider at least once for their last pregnancy, and urban women were more likely than rural women to have received ANC from a skilled provider. Among the total live births in the 5 years preceding the survey, 50% were delivered by a skilled provider and 48% were delivered in a health facility. In the last 2 years preceding survey, 34% of women reported receiving a PNC check-up in the first 2 days after birth [8]. Moreover, female genital mutilation (FGM) is still practiced despite Ethiopia bans medicalization of FGM.

The existence of customary practices and deeply-rooted beliefs that discriminate on the grounds of gender and sexual orientation all testify to a failure to effectively realize sexual and reproductive health rights in Africa [9]. A study conducted in Ethiopia reported various instances of harmful traditional practices during perinatal period such as food prohibition, home delivery, and discarding colostrum. Such harmful traditional practices, beliefs, and taboos are often implicated in determining the care received by mothers during pregnancy and childbirth [10].

SRHR knowledge among rural reproductive-age women is barely studied in Ethiopia. Almost all available studies on knowledge of SRHR in Ethiopia were conducted among female adolescent students in the university, high school or reproductive-age women in urban settings. Those studies revealed that there was low-level SRHR knowledge among participants [11–14].

Sexual and reproductive health rights are inherent entitlements for women. These rights have not been recognized to their maximum potential in Ethiopia, despite the domestication of the international instruments related to their implementation. Because SRHR information is scarce in the study area, this study aimed to determine the magnitude of SRHRs knowledge and use of SRH services and their predictors among rural reproductive-age women in the Aleta Wondo District, Ethiopia.

## Methods

### Study setting, design and period

A community-based cross-sectional study was conducted from April 03 to May 15, 2019. The study was conducted in the Aleta Wondo district, which is found in Sidama Zone, Southern Nations Nationalities and Peoples Region (SNNPR). The district is located 65 km south of Hawassa City and 333 km south of Addis Ababa, the capital city of Ethiopia. At the end of 2018, the aggregate population of the district was 200,593, and the reproductive age women account for 23.3% (46,738) of the district’s total population. The district constitutes 27 rural kebeles (smallest administrative unit in Ethiopia). There were seven public health centers and 27 health posts in the district in 2019.

### Population and sample size determination

All reproductive age group women who were inhabitants of rural kebeles of Aleta Wondo district were a source population. A single population proportion formula was used to determine the sample size for determining the level of sexual and reproductive health rights knowledge and practice. In sample size calculation, 50 % of sexual and reproductive health rights knowledge or practice was taken because of lack of similar study conducted among reproductive age women [15]. Also, we used 5% margin of error, 95% confidence interval, 10% non-response rate and design effect of 2. The sample size calculation yielded a final sample size of 845.

### Sampling procedure

Out of the 27 kebeles found in the district administration, seven kebeles (25%) were selected by a lottery method. The sample was proportionally allocated to the selected kebeles. A systematic random sampling was implemented to select households where reproductive-age women were residing. A list of total households with reproductive-age women present in the family folder of the kebele administration was used as a sampling frame. The family folder contains the list of total households (locally called “Aba-Wora”) in the kebele administration. It is regularly updated and given a number by the administrative bodies through health extension workers of the kebele. For each kebele a sampling interval K was determined. The initial household was selected by a lottery method employing a number between one and K. In case more than one woman in a given household was identified, priority was given to a mother.

### Study variables

**Dependent variables:** sexual and reproductive rights knowledge and sexual and reproductive health services utilization.

**Sexual and reproductive health services utilization:** ever used at least one of the sexual and reproductive health services in healthcare facilities. To assess the SRH services utilization a series of six questions were used. Participants who ever used at least one of SRH services was coded 1 (yes) and never used any of services was coded 0 (No). A woman who had at least one of the sexual and reproductive health services during her life time considered as she had sexual and reproductive health services.

**Sexual and reproductive health rights knowledge:** a series of thirteen knowledge related questions about sexual and reproductive health rights were employed to assess SRHR knowledge [1, 11–14, 16, 17]. Women’s response to each question was coded one for ‘correct’ response and zero for ‘incorrect’ response. Good knowledge of SRHR was defined as equal to or greater than median value of the sum of correct responses Women had poor knowledge of SRHR when the sum correct responses less than the median. Cronbach alpha (α) was calculated to check reliability of knowledge measuring tools. The Cronbach alpha (α) for knowledge was 0.804.

**Independent variables:** age, educational status, religion, occupation, monthly income, educational status of husband, educational status of husband’s mother, occupation of a husband, family income, previous exposure to reproductive health services, having favorable attitude towards SRH services, exposure to SRHR information, access to health facility and health care utilization, social and cultural factors, women’s autonomy and decision making.

### Data collection procedures

An interviewer-administered structured questionnaire was used to gather the data. The questionnaire was first prepared in English and then translated into Sidamigna by language experts and then translated back into English to check completeness and consistency. It consisted of socio-demographic characteristics, sexual and reproductive health services, and information utilization, questions related to sexual and reproductive health rights knowledge and practice and source of information. Seven nurses collected the data, and two nurses supervised the data collection process.

### Data quality assurance

The data collectors and local supervisors were trained for 2 days regarding the objective of the study and how to ask and probe questions during the data collection, and maintaining the confidentiality of the respondents’ information. Before conducting the primary study, a pretest was carried out in two kebeles which were not included in the main study to ensure the validity and reliability of tools. The results of the pretest were discussed, and corrections and changes were made on the questionnaire. The principal investigator and local supervisors supervised the data collection process on a daily basis. At the end of each day, questionnaires were carefully reviewed and checked for completeness, accuracy, and consistency, and corrective measures were undertaken whenever necessary.

### Data analysis

The collected data were entered into EPI INFO version 7.2.2.6 computer software and analyzed using SPSS version 20 statistical program. Descriptive data were presented in frequencies and percentage using Tables. A bivariate analysis was conducted to determine the association between outcome variables and each independent variable. In bivariate analysis, variables whose *P*-value < 0.25 were considered as candidate variables for the multivariable logistic regression model to identify independent predictors by controlling confounding variables.

### Ethical considerations

Ethical clearance was obtained from the Institutional Review Board of College of Medicine and Health Sciences, Hawassa University. Written consent was also obtained from local authorities and concerned government bodies from the Aleta Wondo District Administration. After a brief introduction of the study objective, benefit and possible risk of participation, informed verbal consent was obtained from each study subject and from parents/guardians for participants who were 16–18 years age group. To ensure confidentiality of selected respondents, their official names were not indicated on the structured questionnaire.

## Results

### Socio-demographic and economic characteristics of the participants

**Fig. 1:**
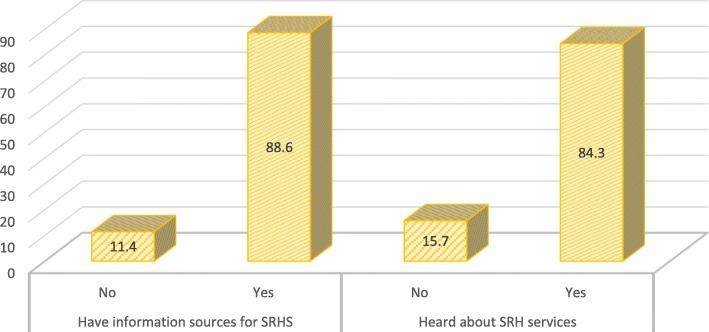
Have source of information and awareness for SRH services among reproductive age women Aleta Wondo woreda, Sidama, 2019(n = 833)

Out of intended 845 study participants, a total of 833 (98.6%) reproductive age women were interviewed. The youngest was 16 years old. The mean age of participants was 29.29 + 6.4 years. More than half, 453(54.4%) of the respondents were from the age group of 25–34 years. The majority (741 or 89.0%) of the participants were married. Regarding ethnicity, 785 (94.3%) of them belong to the Sidama ethnic group. About 81% or 673 respondents were protestant Christians. The majority (536 or 64.3%) of the participants attended formal education (at least a primary level) (Table 1). Participants who had at least one source for RSH services information (either radio, TV, school teacher etc. …) were 89, and 84% of participants had awareness about SRH services (Fig. 1).

**Table 1:** Socio demographic and economic characteristics of reproductive age group women, AletaWondoworeda, Sidama, 2019(*n* = 833)

		Frequency	Percent
Age	15 - 19 yrs	52	6.2
	20-24 yrs	139	16.7
	25-29 yrs	255	30.6
	30-34 yrs	198	23.8
	35-39 yrs	133	16.0
	40-44 yrs	47	5.6
	45-49 yrs	9	1.1
Marital status	Married	741	89.0
	Single	77	9.2
	Widowed	15	1.8
Ethnicity	Sidama	785	94.3
	Amhara	48	5.7
Religion	Orthodox	64	7.7
	Protestant	673	80.8
	Muslim	56	6.7
	Catholic	40	4.8
Educational level	Not attend formal education	297	35.7
	Attended formal education	536	64.3
Income in percentile	Lowest	279	33.5
	Middle	280	33.6
	Highest	274	32.9
Husband’s occupation	Agriculture	385	51.8
	Small Business	206	27.7
	NGO Job	27	3.6
	Daily laborer	56	7.5
	Government Job	69	9.3
Husband’s educational level	Not attend formal education	241	32.4
	Attended formal education	502	67.6
Mother’s in low Educational level	Not attended formal education	643	85.7
	Attended formal education	107	14.3

Mean Age = 29.29 Yrs S.D. = 6.357 Max Age = 49 and Min Age = 16

### Knowledge about reproductive and sexual health rights

The median score was 6.0 out of 13. More than half (56.1%) of participants were found to have poor knowledge. Participants who knew the minimum age of marriage, right to safe abortion and right to have information and services of family planning were 405 (48.6%), 204 (24.5%) and 479 (57.5%), respectively. Participants who knew the right to access to all SRH services without husbands’ permission were 306 (36.7%). Less than half, 326 (39.1%), of the participants knew a presence of legal support for victims of GBV. Half of participants, 419 (50.3%), knew of the right to decide to be free against FGM. Fifty-nine percent of participants didn’t agree with the statement of right to choose the partner, and only 279 (33.5%) of the participants knew about the right to decide whether or not and when to have children (Table 2).

**Table 2:** Responses of the participants to the knowledge related information on reproductive and sexual health right of reproductive age women, AletaWondoworeda, Sidama, 2019(*n* = 833)

Characteristics		Frequency	Percent
Know legal minimum age of marriage	No	428	51.4
	Yes	405	48.6
Know right to have safe abortion and post abortion care at health facility	No	629	75.5
	Yes	204	24.5
Know right to have information and services of family planning method	No	354	42.5
	Yes	479	57.5
Know the full right to access for all SRH services without husbands will	No	527	63.3
	Yes	306	36.7
Know the right of confidential and privacy at health facility	No	416	49.9
	Yes	417	50.1
Know every woman has the right of protection from sexual abuse, reproductive harms and sexual discrimination	No	380	45.6
	Yes	453	54.4
Know the right of Legal support for victims of GBV	No	507	60.9
	Yes	326	39.1
Know the right of adolescents	No	347	41.7
	Yes	486	58.3
Know the right to decide to be free of against FGM	No	414	49.7
	Yes	419	50.3
Know the right to have information and education	No	235	28.2
	Yes	598	71.8
Know the right to choose the partner	No	497	59.7
	Yes	336	40.3
Know the right to have consensual sexual relations	No	457	54.9
	Yes	376	45.1
Know the right to decide whether or not and when to have children	No	554	66.5
	Yes	279	33.5
	Total	833	100.0

Mode = 2.00 Median = 6.0000 Mean = 6.1032,

Poor knowledge = 467(56.1%) Good knowledge = 366(43.9%)

### Utilization of sexual and reproductive health services

Less than half of the participants, 384 (46.1%), had been counseled on harmful traditional practices (HTP) including FGM. Among participants who had a history of pregnancy and childbirth, 554 (66.5%) delivered in health facility. Also 605 (72.6%), and 436 (52.3%) had ANC and PNC services in health facility respectively. Participants who ever used family planning services and HIV counselling were 657 (78.9%) and 453 (54.4%), respectively. Moreover, 145(17.4%) of the participants had counseled and treatment for STI and 64 (7.7%) participants had safe abortion care at health facilities (Table 3).

**Table 3:** Responses of the participants to the Practice related information on reproductive and sexual health right of reproductive age women, AletaWondoworeda, Sidama, 2019(*n* = 833)

Characteristics		Frequency	Percent
Ever used family planning methods	No	176	21.1
	Yes	657	78.9
Ever used ANC service	No	228	27.4
	Yes	605	72.6
Ever used delivery services at health facilities	No	279	33.5
	Yes	554	66.5
Ever used PNC services at health facilities	No	397	47.7
	Yes	436	52.3
Ever had education on HTP including FGM	No	449	53.9
	Yes	384	46.1
Ever had HIV counseling and testing services	No	380	45.6
	Yes	453	54.4
Ever had STIs counselling and treatment services	No	688	82.6
	Yes	145	17.4
Ever had legal abortion and post abortion care	No	769	92.3
	Yes	64	7.7

### Predictors of sexual and reproductive health rights knowledge

Independent variables which were associated with SRHR knowledge at binary logistic regression analysis were women’s educational status, household monthly income, husband’s occupation, husband’s educational status, mother-in-law’s educational status, having at least one of the SRHR information sources and knowing SRH services and providing institutions. In multivariate logistic regression analysis, women’s educational status, household monthly income, having at least one SRHS information source and knowing SRH services and providing institutions were found to be independent predictors of sexual and reproductive health rights knowledge. Participants who had formal education were about 27 times more likely to be knowledgeable than the participants who didn’t attend formal education [AOR: 27.812, 95%CI: 13.650, 56.669]. Participants who had the highest percentile of monthly income were four times more likely to be knowledgeable than participants who had the lowest percentile of monthly income [4.048, 95%CI: 2.432, 6.736]. Participants who had at least one source of information on SRHR were four times more likely to be knowledgeable than participants who didn’t have information sources [4.339, 95%CI: 1.236, 15.232]. Participants who knew about SRH services and providing institutions were nine times more likely to be knowledgeable than participants who didn’t know about SRH services [AOR: 9.158,95% CI: 3.390,24.738] (Table 4).

**Table 4:** Bivariate and multivariable logistic regression analysis output for SRH rights knowledge among reproductive age group women AletaWondo, Sidama 2019

Variables		Knowledge		Crude OR (95%CI	Adjusted OR (95%CI
		Poor knowledge	Good knowledge		
Educational status	No formal education	279 (93.9%)	18 (6.1%)	1	
	Formal education	188 (35.1%)	348 (64.9%)	28.691 (17.254,47.712)	27.812 (13.650,56.669)
Income percentile	Lowest	198 (71.0%)	81 (29.0%)	1	
	Middle	188 (67.1%)	92 (32.9%)	1.196 (0.835,1.713)	5.8 (3.276,10.273)
	Highest	81 (29.6%)	193 (70.4%)	5.824 (4.038,8.401) ^**^	4.048 (2.432,6.736) ^***^
Husband’s occupation	Agriculture	282 (73.2%)	103 (26.8%)	1	
	Small Business	85 (41.3%)	121 (58.7%)	3.897 (2.726,5.573) ^**^	3.629 (1.391,9.469)
	NGO Job	5 (18.5%)	22 (81.5%)	12.047 (4.445,32.645) ^**^	2.015 (0.767,5.296)
	Daily laborer	29 (51.8%)	27 (48.2%)	2.549 (1.441,4.510) ^**^	1.115 (0.260,4.774)
	Government Job	12 (17.4%)	57 (82.6%)	13.005 (6.707,25.216) ^**^	2.63 (0.837,8.274)
Husband’s education	No formal education	198 (82.2%)	43 (17.8%)	1	1
	Formal education	215 (42.8%)	287 (57.2%)	6.147 (4.228,8.936) ^**^	0.902 (0.488,1.667)
Mother’s in laws educational	No formal education	388 (60.3%)	255 (39.7%)	1	
	Formal education	29 (27.1%)	78 (72.9%)	4.092 (2.597,6.448) ^**^	1.198 (0.673,2.131)
Have SRHS information sources	No	84 (88.4%)	11 (11.6%)	1	
	Yes	383 (51.9%)	355 (48.1%)	7.078 (3.714,13.488) ^**^	4.339 (1.236,15.232) ^***^
Know SRH services and providing institutions	No	116 (88.5%)	15 (11.5%)	1	
	Yes	351 (50.0%)	351 (50.0%)	7.733 (4.427,13.508) ^*^	9.158 (3.390,24.738) ^***^

Key:- At bivariate, *p* < 0.25 ^**^ significant at multivariate, *p* < 0.05^***^

### Predictors of sexual and reproductive health services utilization

In binary logistic regression analysis, maternal age, marital status, women’s educational status, household’s monthly income, husband’s occupation, husband’s educational status, mother-in- law’s educational status, having sexual and reproductive health information sources and knowing about SRH services and providing institutions were significantly associated with utilization of SRH services. In multivariate logistic regression analysis, women’s educational status, household’s monthly income, and knowing about SRH services and providing institutions were independent predictors of SRH services utilization. Participants who had formal education were about 5 times more likely to utilize SRH services than participants who didn’t have formal education [AOR: 4.807, 95%CI: 2.899, 7.968]. Participants who had highest monthly income were two times more likely to utilize SRH services than participants who had the lowest monthly income, [AOR: 2.223, 95%CI: 1.475, 3.349], and participants who knew about SRH services and providing institutions were four times more likely to utilize SRH services than participants who did not know SRH services and providing institutions [AOR: 4.012, 95%CI: 1.881, 8.560] (Table 5).

**Table 5:** Bivariate and multivariable logistic regression analysis output SRH services utilization among reproductive age group women AletaWondo Sidama 2019

Variables		Practice		Crude OR 95%CI	Adjusted OR (95%CI
		Never practiced	Ever practiced		
Age	15-19 yrs	48 (92.3%)	4 (7.7%)	1	
	20-24 yrs	75 (54.0%)	64 (46.0%)	10.240 (3.501,29.947) ^**^	0.23 (0.012,4.323)
	25-29 yrs	142 (55.7%)	113 (44.3%)	9.549 (3.343,27.275) ^**^	0.165 (0.019,1.446)
	30-34 yrs	130 (65.7%)	68 (34.3%)	6.277 (2.172,18.142) ^**^	0.207 (0.024,1.776)
	35-39 yrs	88 (66.2%)	45 (33.8%)	6.136 (2.081,18.095) ^**^	0.0292 (0.034,2.516)
	40-44 yrs	34 (72.3%)	13 (27.7%)	4.588 (1.377,15.290) ^**^	0.203 (0.023,1.775)
	45-49 yrs	8 (88.9%)	1 (11.1%)	1.500 (0.148,15.197)	0.341 (0.036,3.225)
Marital status	Married	438 (59.1%)	303 (40.9%)	1	
	Single	76 (98.7%)	1 (1.3%)	0.019 (0.003,0.138) ^**^	0.912 (0.524,1.892)
	Widowed	11 (73.3%)	4 (26.7%)	0.526 (0.166,1.666)	
Educational status	No formal education	254 (85.5%)	43 (14.5%)	1	
	Formal education	271 (50.6%)	265 (49.4%)	5.776 (4.010,8.320) ^**^	4.807 (2.899,7.968) ^***^
Income in percentile	Lowest	230 (82.4%)	49 (17.6%)	1	
	Middle	189 (67.5%)	91 (32.5%)	2.260 (1.519,3.362) ^**^	
	Highest	106 (38.7%)	168 (61.3%)	7.439 (5.023,11.017) ^**^	2.223 (1.475,3.349) ^***^
Husband’s occupation	Agriculture	272 (70.6%)	113 (29.4%)	1	
	Small Business	101 (49.0%)	105 (51.0%)	2.502 (1.763,3.552) ^**^	1.676 (0.855,3.289)
	NGO Job	7 (25.9%)	20 (74.1%)	6.877 (2.829,16.718)	1.364 (0.700,2.656)
	Daily laborer	38 (67.9)	18 (32.1%)	1.140 (0.624,2.082)	0.779 (0.262,2.317)
	Government Job	21 (30.4%)	48 (69.6%)	5.502 (3.150,9.610) ^**^	2.678 (1.119,6.409)
Husband’s Education	No formal education	184 (76.3%)	57 (23.7%)	1	
	Formal education	255 (50.8%)	247 (49.2%)	3.127 (2.215,4.414) ^**^	0.851 (0.514,1.408)
Mother’s in laws education	No formal education	404 (62.8%)	239 (37.2%)	1	
	Formal education	40 (37.4%)	67 (62.6%)	2.831 (1.855,4.322) ^**^	1.254 (0.769,2.047)
Have SRHS information sources	No	84 (88.4%)	11 (11.6%)	1	
	Yes	441 (59.8%)	297 (40.2%)	5.143 (2.697,9.807) ^**^	1.040 (0.441,2.449)
Know SRH services and providing institutions	No	118 (90.1%)	13 (9.9%)	1	
	Yes	407 (58.0%)	295 (42.0%)	6.579 (3.640,11.893) ^**^	4.012 (1.881,8.560) ^***^

Key:- at bivatiate, *p* < 0.25 ^**^ significant at multivariate, *p* < 0.05^***^

## Discussion

In this study 43.9% of respondents had good knowledge of sexual and reproductive health rights. The finding of our study was lower than reports from various studies conducted in Ethiopia; Wolayta Sodo University, Shire Town Tigray, Northern, Asella Town, and Adet Tana Haik College [11–14] and a study conducted among married women in Nepal [18]. The potential difference could be due to differences in study population and study site. In our study, rural women were respondents, unlike other studies conducted in Ethiopia among university or college students.

Less than half (48.6%) of participants knew the minimum age of lawful marriage. This figure was lower than studies conducted in Nepal [16, 17]. This could be due to difference in the study setting, socio-economic, and education status of participants and access to SRHR information sources. Participants who knew about the right to access to all SRH services without husbands’ permission were 36.7%. This is may be due to male dominance in decision making about utilization of health services.

In this study women from household with the highest monthly income and those with formal education and with sources of information were more knowledgeable than others. This finding is consistent with the studies conducted at Shire Town [12], in Oromiya, Asella town [13] and the study from Nepal [18]. Also a study conducted in Bangladesh revealed that the respondents, who were literate, were more aware of their reproductive health rights [19]. This could be due to the fact that higher socioeconomic status is typically associated with better health, including better sexual and reproductive health awareness and practices, which could be attributed to better access to information. Participants who had formal education and attaining higher educational status enhances women’s ability to access information and services to exercise more control over their reproductive lives [20, 21]. In some settings women’s higher educational attainment is associated with improved access to health care, fewer births, healthier and better educated children than women with lower educational status [22]. Education can empower women with a new vision and normative orientation, better health care, better employment opportunities outside home, and better knowledge of access to SRH services [20].

Regarding SRH services utilization, it was found that 37.0% of the respondents had ever had SRH services, a lower figure than in Nepal [18], in Mizan Tepi (65%) and Nekemte town in Ethiopia [23, 24]. This was due to differences in the study settings and study population. Other studies were conducted among female university or college students, who had more exposure to SRH services.

In this study, respondents who had formal education, those from households with the highest income, and those who knew about reproductive health services were more likely to utilize SRH services as compared to their counterparts. This figure was similar to the finding of 2016 demographic and health survey, and other studies conducted in Ethiopia [8, 24, 25]. This could be due to the fact that a more educated segment of population may have better information about the services and those with the highest monthly income may have improved access to different sources of information such as radio, newspaper or other print and mass media and which, in turn, could contribute to increasing demand for health services.

The cross-sectional design of the study did not permit a determination of the temporal relationship between dependent and independent variables. Various scientific procedures were undertaken during tool preparation, study participants selection, data collection and analysis to reduce bias and control confounders.

## Conclusion

Both knowledge and practice of reproductive and sexual health rights are enormously important to achieve HSTP of Ethiopia and the targets of health related SDGs. In this study context specific factors, such as education and household monthly income were identified as predictors for knowledge of SRHR and SRH services utilization. Stakeholders and government should conduct programs to promote women’s educational status and household economic status to enhance women’s SRHR Knowledge and SRH services utilization.

## Supplementary information

**Additional file 1.** English language questionnaire.

## Data Availability

The data will be available from the corresponding author upon justifiable requests.

## Abbreviations

ANC: Antenatal Care
EDHS: Ethiopian Demographic and Health Survey
FGM: Female Genital Mutilation
HIV: Human immunodeficiency virus
HSTP: Health Sector Transformational Plan
HTP: Harmful Traditional Practices
ICPD: International Conference on Population and Development
PNC: Postnatal Care
SDG: Sustainable Development Goals
SRH: Sexual and Reproductive Health
SRHR: Sexual and Reproductive Health and Rights
WHO: World Health Organization

## References

[1] UN Population Fund (2004). Programme of action adopted at the international conference on population and development, Cairo, Sept 5–13, 1994.

[2] UN Population Fund, Center for Reproductive Rights (2013). ICPD and human rights: 20 years of advancing reproductive rights through UN treaty bodies and legal reform.

[3] Serra S (2014). ICPD beyond 2014: moving beyond missed opportunities and compromises in fulfillment of sexual and reproductive health and rights. Glob Public Health.

[4] UNFPA (2010). Sexual and reproductive health for all- reducing poverty, advancing development and protecting human rights.

[5] Federal Democratic Republic of Ethiopia Ministry of Health (2006). National reproductive health strategy 2006–2015.

[6] The Federal Democratic Republic of Ethiopia-Constitution of the Federal democratic Republic of Ethiopia, 1995.

[7] The Federal Democratic Republic of Ethiopia Ministry of Health- Health Sector transformational plan-HSTP 2015/16–2019/20 (2008–2012 EFY) October 2015.

[8] Ethiopian Public Health Institute (EPHI) [Ethiopia] and ICF (2019). Ethiopia mini demographic and health survey 2019: key indicators.

[9] Pretoria University Law Press (2017). Reproductive and sexual rights in sub-Saharan African courts, volume III.

[10] Gedamu H, Tsegaw A, Debebe E (2018). The prevalence of traditional malpractice during pregnancy, child birth, and postnatal period among women of childbearing age in meshenti town, 2016. Int J Reprod Med.

[11] Yohannes MA, Abebaw GW, Zelalem BM (2013). Knowledge of reproductive and sexual rights among university students in Ethiopia. BMC Int Health Hum Rights.

[12] Gebretsadik GG, Weldearegay GG (2016). Knowledge on reproductive and sexual rights and associated factors among youths, Shire town, Tigray, Northern Ethiopia. Int J Res Pharm Sci.

[13] TigistTafa (2015). Assessment of reproductive health right knowledge and practice among preparatory school female students of Asellatown,Oromiya region in Ethiopia:Addis Ababa university school of public health.

[14] Mulatu A, Dabere N, Getachew S, Ayal D (2019). Knowledge and attitude towards sexual and reproductive health rights and associated factors among Adet Tana Haik college students, Northwest Ethiopia. BMC Res Notes.

[15] Sullivan L. Power and sample size determination. http://sphweb.bumc.bu.edu/otlt/MPH-Modules/BS/BS704_Power/BS704_Power_print.html. Accessed on 7 Feb 2020.

[16] Yadav RK (2016). Knowledge and practice on reproductive heath rights among married women in Nepal. JHAS.

[17] Kaphle M (2013). Awareness and utilization of reproductive rights among the women of reproductive age in Kapan VDC, Nepal. JHAS.

[18] Yadav RK et.al. Knowledge and practice on reproductive heath. JHAS, 2016, Vol. 5, No. 1 P 53–57.

[19] Hossain MK, Mondal MNI, Akter MN (2011). Reproductive health rights of women in the rural areas of Meherpur District in Bangladesh. J Reprod Infertil.

[20] Jejeebhoy S (1995). Women’s education, autonomy and reproductive behavior: experience from developing countries.

[21] WHO (2014). Maternal mortality, to improve maternal health, barriers that limit access to quality maternal health services must be identified and addressed.

[22] Güneş PM (2015). The role of maternal education in child health: evidence from a compulsory schooling law. Econ Educ Rev.

[23] Yayehyirdah Y, Rediet G, Matewal Y, Melkamsew A, Kifle A, Alemayehu A, Fuad N (2017). Assessment of knowledge, attitude and practice to ward reproductive health services among MizanTepi University TepiCampass students, Sheka Zone Ethiopia. IMedpub J.

[24] Wakgari B, Taklu M, Mulusew G, Melese S (2018). Sexual and reproductive health services utilization and associated factors among secondary school students in Nekemte town. Reprod Health J.

[25] Atitegeb A, Teketo K, Getachew H (2016). Level of young people sexual and reproductive health service utilization and its associated factors among young people in Awabel District, Northwest Ethiopia. PLoS One.

