# Supplementary material for: Sexual and reproductive health rights knowledge and reproductive health services utilization among rural reproductive age women in Aleta Wondo District, Sidama zone, Ethiopia: community based cross-sectional study
Source: BMC Int Health Hum Rights. 2020 Mar 11;20:4. doi: 10.1186/s12914-020-00223-1 (PMC7065338; doi:10.1186/s12914-020-00223-1)
Supplement: Supplementary file 1 — Additional file 1. English language questionnaire. [file 12914_2020_223_MOESM1_ESM.docx]

## English version questionnaire

**Part I: Socio-demographic characteristics of the participants**

| **No.** | **Question** | **Choices** | **Remark** |
| --- | --- | --- | --- |
| 1 | What is the name of your kebele? | 1. -------------------------- |  |
| 2 | How old are you?(age in years) | Enter# _______________ |  |
| 3 | What is your marital status? | 1. Married 2. Single 3. Widowed 4. Divorced |  |
| 4 | What is your ethnicity? | 1. Sidama 2. Gurage 3. Wolayta 4. Amahara 5. Others (**specify**)_______________ |  |
| 5 | What is your religion? | 1. Orthodox 2. Protestant 3. Muslim 4. Catholic   5. Others (**specify**)_________________ |  |
| 6 | What is your educational level? | 1 can't read and write  2 can Read and write  3 1-6 4 7-12  5 diploma and Above |  |
| 7 | What is your monthly income? | ___________Ethio.birr |  |
| 8 | What is your Place of residence? | 1. Rural  2. Urban |  |
| 9 | How old is your huband?(age in years) | Enter# _______________ |  |
| 10 | What is your husband’s occupation? | 1. Agriculture 2. Small business 3. NGO Job 4. Daily labourer   5 Government Job |  |
| 11 | What is your husband’s educational level? | 1 can't read and write  2 can Read and write  3 1-6   1. 7-12   5 diploma and Above |  |
| 12 | What is your husband’s monthly income? | ___________Ethio. Birr |  |
| 13 | What is your mother’s in low educational level? | 1. can't read and write   2 can Read and write  3 1-6 4 7-12  5 diploma and Above |  |

**Part II. Sources of information for SRHRs & practice and Knowledge related information of service utilization.**

| 14 | | Do you have sources of information for reproductive and sexual health services and rights? | 1. Yes 2. No | Go Q15 Go Q16 | | |
| --- | --- | --- | --- | --- | --- | --- |
| 15 | | If “**Yes”** to **Q 14**, what is your source of information? ( More than one response is possible) | 1. Television 2. Radio 3. Newspaper 4. School massmediya 5. School teachers 6. Prior school health club 7. Family member 8. Heath care provider 9. Friends 10. Community discussion 11. Specify any other____________________ |  | | |
| 16 | | If “**NO”** to **Q 14**, Do you think that it is important for reproductive health services of Women? | 1. Yes 2. No |  | | |
| 17 | | Have you ever trained about reproductive health services? | 1. Yes 2. No |  | | |
| 18 | Do you know about sexual & reproductive health services? | | 1. Yes 2. No | Gt to Q 19& 20 | | |
| 19 | If “**Yes”** to **Q 18 above**, specify service delivery points in your woreda ?( More than one response is possible) | | 1. Public health post 2. Public health centre 3. Public hospital 4. Private health institution 5. Nongovernmental institution |  | | |
| 20 | If **Yes”** to **Q 18 above**, what services is/are provided there do you Know?( More than one response is possible) | | 1. Counselling about reproductive and sexual health services including VAW 2. Family planning provision 3. Antenatal care services 4. Delivery services 5. Postnatal care services 6. About the danger of harmful traditional services including FGM/C 7. HIV counselling and test 8. STIs prevention and treatment 9. Legal abortion and post abortion care 10. Specify any other---------------------------- |  | | |
| 21 | The proper utilization and frequency of ANC Service is? | | 1. 1time 2. 2 times 3. 3 times 4. 4 times or more |  | | |
| 22 | \| How do you rate the risk of not using those services \| \| --- \| | | 1. Not risky 2. Low risk 3. Moderate risk 4. High risk 5. I do not know |  | | |
| **Part III. Knowledge related information of reproductive and sexual health right** | | | | | | |
| 23 | Do you know reproductive and sexual health rights are the parts of components of human Rights | | 1. Yes 2. No | | Go Q24 -38 Go Q50 | |
| 24 | Do you know legal minimum age of marriage (-------) (ask and fill in number) | | 1. Yes 2. No | |  | |
| 25 | Do you know right to have safe abortion and post abortion care at health facility | | 1. Yes   2 No | |  | |
| 26 | Right to have information and services of family planning method of choice | | 1. Yes 2. No | |  | |
| 27 | Do you know the full right to access for all RH and sexual health services without husband’s will | | 1. Yes 2. No | |  | |
| 28 | Do you know the right of confidential and privacy health services | | 1. Yes 2. No | |  | |
| 29 | Do you know everywoman has the right of protection from sexual abuse, reproductive harms and sexual discrimination? | | 1. Yes 2. No | |  | |
| 30 | Do you know Legal support for victims of GBV? | | 1. Yes 2. No | |  | |
| 31 | Do you know the right of adolescents? | | 1. Yes 2. No | |  | |
| 32 | Do you know the right to decide to be free against FGM? | | 1. Yes   2 No | |  | |
| 33 | Do you know the right to have information and education? | | 1. Yes   2 No | |  | |
| 34 | Do you know the right to choose the partner? | | 1. Yes   2 No | |  | |
| 35 | Do you know the right to have consensual sexual relations? | | 1. Yes   2 No | |  | |
| 36 | Do you know the right to have consensual marriage? | | 1. Yes   2 No | |  | |
| 37 | Do you know the right to decide whether or not, and when, to have children | | 1. Yes   2 No | |  | |
| 38 | Do you know the right to have pursue a satisfying, safe and pleasurable sexual life | | 1. Yes   2 No | |  | |
| **Part IV**: - **Sexual and reproductive health service utilization related questions** | | | | | | |
| 39 | | Have you ever used family planning methods? | 1. Yes 2. No | | |  |
| 40 | | Have you ever counselled about reproductive and sexual health services including VAW? | 1. Yes   2. No | | |  |
| 41 | | Have you ever used antenatal care services? | 1. Yes   2. No | | |  |
| 42 | | Have you ever used delivery services at health facilities? | 1. Yes 2. No | | |  |
| 43 | | Have you ever used postnatal care services at health facilities? | 1. Yes 2. No | | |  |
| 44 | | Have you ever counselled about the danger of harmful traditional services including FGM/C | 1.Yes  2. No | | |  |
| 45 | | Have you ever used HIV counselling and test services? | 1.Yes  2.No | | |  |
| 46 | | Have you ever used STIs prevention and treatments services? | 1. Yes  2.No | | |  |
| 47 | | Have you ever utilized legal abortion and post abortion care | 1. Yes  2.No | | |  |
| 48 | | If you ever used at least one of services listed above in questions “40-47**”**, who decide for you to go to those services? | 1. Only Husband 2. You your self 3. Mother in law | | |  |
| 49 | | What do you do if your daughter becomes pregnant from her relative by raping before marriage and marriage age? ( More than one response is possible ) | 1. I never word to anybody 2. I will tell to my husband and then go to justice bodies 3. I will take to abortion service traditional 4. I will take to safe abortion services at health facility 5. Such things could not be occurred | | |  |
